# Benefits of Regular Table Tennis Practice in Body Composition and Physical Fitness Compared to Physically Active Children Aged 10–11 Years

**DOI:** 10.3390/ijerph18062854

**Published:** 2021-03-11

**Authors:** Francisco Pradas, Ignacio Ara, Víctor Toro, Javier Courel-Ibáñez

**Affiliations:** 1 Research Group Training, Physical Activity and Sports Performance (ENFYRED), University of Zaragoza, 22003 Huesca, Spain; franprad@unizar.es; 2 Research Group Growth, Exercise, Nutrition and Development (GENUD), Toledo Research Group, Universidad de Castilla-La Mancha, 45071 Toledo, Spain; ignacio.ara@uclm.es; 3 Department of Physiology, Faculty of Sports Science Faculty, University of Extremadura, University Avenue, 10003 Cáceres, Spain; vtororom@alumnos.unex.es; 4 Department of Physical Activity and Sport, Faculty of Sport Sciences, University of Murcia, 30720 Murcia, Spain

**Keywords:** healthy growth, childhood, exercise, leisure-time physical activity, racket sport

## Abstract

The aim of this study was to identify the differences in body composition and physical fitness between children who played table tennis regularly during a two-year period compared to physically active children who were not engaged in a regular activity. Three hundred seventy-four children aged 10 to 11 years were divided into two groups: table tennis players (*n* = 109 boys and 73 girls) and physically active group (*n* = 88 boys and 104 girls). Anthropometric analysis included body mass index, skinfolds, perimeters and bone diameters. Somatotype and body composition were determined according to age-specific equations. Physical fitness assessment included hand grip dynamometry (strength), sit-and-reach test (range of movement) and maximal multistage 20 m shuttle run test (cardiovascular fitness). The result show that children who regularly played table tennis had greater bone development and superior physical fitness compared to those who were physically active but not engaged in a regular physical activity. This is the largest study to date presenting data about the potential of table tennis to benefit health in children. These results constitute an important first step in clarifying the effectiveness of table tennis as a health-promotion strategy to encourage children to undertake regular physical activity and limit sedentary behavior.

## 1. Introduction

Adherence to regular physical activity throughout childhood promotes healthy growth and development [1]. Evidence indicates that physically active children are likely to maintain a healthy lifestyle in adulthood [2]. Lifelong regular exercise contributes to living longer and to better cardiometabolic and cognitive conditions [3,4], which delay the onset of 40 chronic diseases [5,6,7]. Among the options for exercise, racket sports, and particularly table tennis, stand as a universal practice for children and adults to have fun, improve physical fitness, and develop motor and cognitive skills [8,9,10]. Additionally, recent studies suggest that table tennis may also be a particularly effective activity for promoting health and increasing leisure-time physical activity among sedentary populations [11,12]. This is relevant in todays’ world, considering that insufficient physical activity has raised the level of unhealthy body composition, e.g., overweight and obesity are at epidemic proportions with an alarming increase of ten-fold over the last forty years [13]. Interestingly, while natural sex differences in body composition and physical fitness could exist during childhood [14,15], increments in cardiovascular fitness through exercise positively contribute to reducing excess weight and cardiometabolic risk factors (body mass index, waist to hip ratio, and fat mass index) in adolescents, regardless of sex [16]. Accordingly, table tennis can be an effective strategy to encourage children and adolescents to undertake regular physical activity for optimal health outcomes and to limit sedentary behavior, particularly recreational screen time [17].

The popularity of table tennis has constantly increased since it became an Olympic sport in the 1990s, reaching over 300 million practitioners worldwide [18]. The rising fame of table tennis can be attributed to its intermittent and explosive nature, with highly frequent and intense actions that take place around a small table of 2.74 × 1.52 m^2^ [19,20]. Players are required to hit the ball over 30 times per minute during rallies no longer than 4 s, with short resting times of less than 15 s [19]. These characteristics make table tennis a very intense sport, with the ball travelling at a high speed (>50 km·h^−1^), forcing players to respond in milliseconds [20]. Consequently, agility, reaction time, ballistic strength, and coordination are essential skills than can be developed by regular table tennis practice [21]. Indeed, among older people, table tennis greatly stimulates cognitive function as compared to other exercises [22]. Additionally, benefits of table tennis practice in muscle strength and neuromotor skills are shown to be maintained in older people [11,23].

Energy demands in competitive table tennis rely on anaerobic glycolysis and triphosphate–phosphocreatine (ATP-PCr) pathways during maximal short-duration efforts, in conjunction with the aerobic system during recovery [19,20]. More specifically, previous studies reported low blood-lactate concentrations (<2.5 mmol·L^−1^) after a competitive table tennis game [19], suggesting that work–rest ratio is sufficiently balanced to avoid glycogen depletion and reaching the sensation of discomfort accompanying muscle fatigue [24,25]. Because of these particular physical demands, table tennis appears to contribute to maintaining a healthy body composition (i.e., appropriate amount of body fat, lean muscle mass, and bone health) among regular adult practitioners [26]. While there are no data available on children, it can be expected that regular table tennis practice twice a week constitutes an enjoyable activity that would contribute considerably to satisfying the recommended level of moderate to vigorous physical activity in children [17].

Despite the potential benefits of table tennis in promoting physical activity from childhood on and its likely influence on a healthier adulthood, the effects of regular table tennis on children’s wellbeing are still unclear. The aim of this study was to identify the differences in body composition and physical fitness between boys and girls aged 10–11 years who played table tennis regularly during two years, compared to physically active children who were not engaged in a regular activity.

## 2. Materials and Methods

### 2.1. Participants

Three hundred seventy-four children aged 10–11 years volunteered to participate in the study. Participants were divided into two groups: table tennis players (*n* = 109 boys and 73 girls) and physically active group (*n* = 88 boys and 104 girls). Table tennis players were recruited from sport clubs and required to meet the following criteria: (i) to have played table tennis as their unique regular physical activity and (ii) to have maintained a training routine of at least 5 h·wk^−1^ over two years. Physically active children were recruited from primary schools and included based on the following criteria: (i) to have practiced no regular physical activity or sports during the last two years in a federative club and (ii) to have been physically active (Physical Activity Questionnaire Short Form (IPAQ-SF) > 1000 min·wk^−1^). Additionally, all participants were required to: (a) have not suffered from diseases; (b) have had no medical treatment or supplementation; and have had no modification in their nutritional habits or physical activity during the last year. After both comprehensive verbal and written explanations of the study, written informed consent was obtained from parents or legal tutors. The Ethics Committee of the University of Zaragoza (ID:19/2010) reviewed and approved the study.

### 2.2. Anthropometric Measurements

Body composition and physical fitness evaluations were carried out under laboratory conditions at the same time of day. Anthropometric analysis was conducted following the International Society for the Advancement of Kinanthropometry (ISAK) procedures [27]. Body mass (kg) and height (m) were collected using a scale (Seca 769, Seca, Hamburg, Germany) and a measuring rod (Seca 220, Seca, Hamburg, Germany) with an accuracy of ±0.001 kg and 0.001 m. Body mass index (BMI) was calculated from body mass (kg) and stature (m^2^) relationship. Skinfolds (triceps, subscapular, supra-iliac, abdominal, thigh, and medial calf) were assed using a skinfold compass accurate to ±0.2 mm (Holtain 610ND, Holtain, Crymych, UK). Perimeters (arm relaxed and calf) and bone diameters (Bistyloid, biepicondylar humerus and biepicondylar femur breadths) were obtained with a bone diameter compass (Holtain 604, Holtain, Crymych, UK) and a brand tape (Seca 201, Seca, Hamburg, Germany) accurate to ±1.0 mm. All anthropometric measurements were taken on the left body side, and all measurements were made by the same operator. Three readings to the nearest 0.5 mm were taken at each skinfold site and the average value was retained for analysis. Somatotype (ectomorph, endomorph, and mesomorph) was determined according to Carter and Heath [28]. Body composition was estimated from specific equations [29,30,31,32,33,34] to calculate fat mass, lean mass, and bone density.

### 2.3. Physical Fitness

Participants were familiarized with the physical fitness tests before data collection. Full-body anthropometry and body composition assessment were first conducted. The physical fitness test started after 15 min of standardized warm-up in the following order: hand grip strength, sit-and-reach test, and maximal multistage 20 m shuttle run test. Handgrip strength was measured using a dynamometer Takei 5101 (Takei Instruments Ltd., Tokyo, Japan). Participants performed two maximal voluntary contractions with the dominant hand and the arm completely extended. The hilt of the dynamometer was adjusted to the participants’ hands [35]. The best of two alternative repetitions was chosen. The sit-and-reach test was used to measure the range of motion (ROM) of the lower back and hamstring muscle, according to standardized procedures [36,37]. From a seated position on the floor with the legs fully extended, participants reached forward along the measuring scale as far as possible without bending the knee, placing one hand on top of the other with palms down. The best of two repetitions was chosen. Cardiovascular fitness was examined by a maximal multistage 20 m shuttle run test [38]. Sound signals were emitted from a pre-recorded tape that increased 0.5 km·h^−1^ each minute from a starting speed of 8.5 km·h^−1^. When the subject could no longer follow the pace, the last stage number announced was used to estimate the maximal oxygen uptake (VO_2_ max) by formula (1) specified in the literature [39]. Absolute VO_2_ max (L·min^−1^) was computed from the resulting relative values.

VO_2_max (mL·kg^−1^·min^−1^) = 31.025 + 3.238·speed − (3.248·age) + (0.1536·speed·age) (1)

### 2.4. Statistical Analysis

Means, standard deviations (SD), and range (min–max) were computed. The Kolmogorov–Smirnov test and Q–Q plots were used to determine the normal distribution of the variables. Homogeneity of the variances was examined by the Levene test. A two-way ANOVA (group effect, sex effect and interaction group × sex effect) was used to identify differences in anthropometry, body composition and physical fitness between the groups of children. Means difference (MD) and 95% CI were calculated for comparison between boys in the group of table tennis players and boys in the physically active group, and the same for girls who play table tennis and active girls. Effect size (ES) was calculated using the Hedge’s *g* test of unequal samples, interpreted as small (0.20), medium (0.50) and large (0.80). The level of significance was set at *p* < 0.05. Power analysis was conducted with G*Power 3.1.9.7 software [40]. Statistical analyses were carried out with IBM SPSS Statistics 22.0 for Windows (IBM Corp., Armonk, NY, USA).

## 3. Results

**Figure 1:**
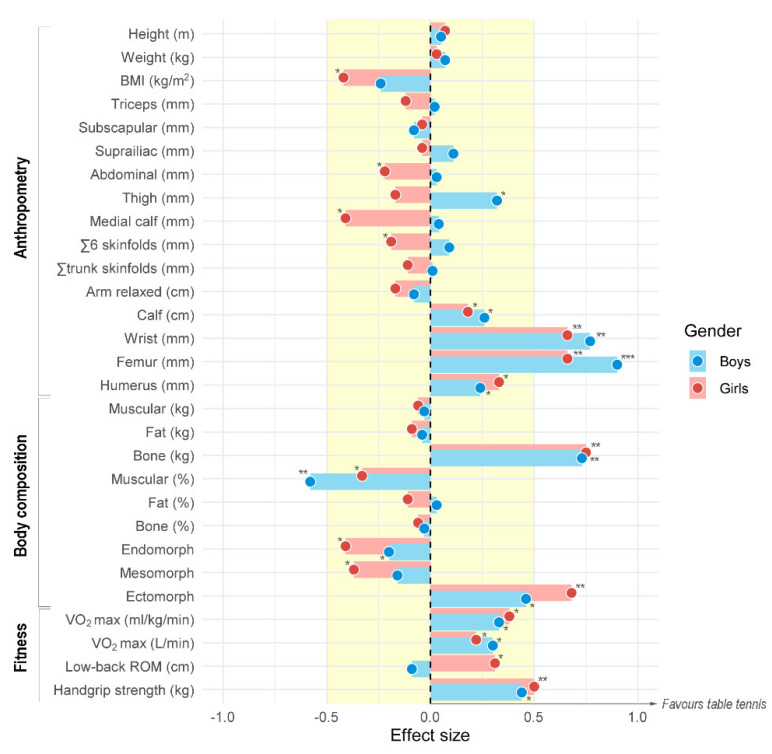
Forest plot showing the effect sizes of the differences in anthropometry, body composition, and physical fitness between boys and girls aged 10–11 years who regularly practice table tennis against those who are physically active but not engaged in a regular activity. Clear markers (red) are girls, dark markers (blue) are boys. Significant differences (*p* < 0.05): * effect size (ES) ≥ 0.20, ** ES ≥ 0.50, *** ES ≥ 0.80.

**Figure 2:**
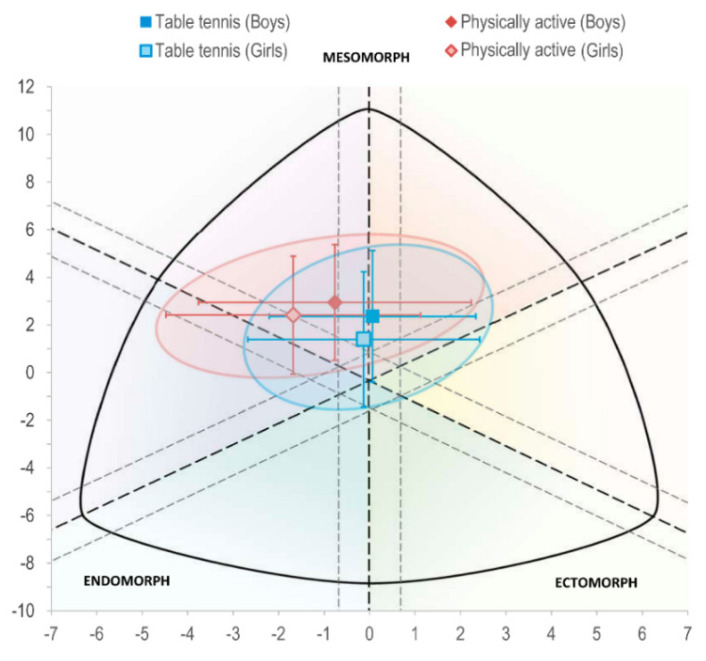
Somatochart of boys and girls aged 10–11 years who regularly practice table tennis against those who are physically active but not engaged in a regular activity. Error bars are standard deviations.

Power analysis determined that the current sample size (*n* = 374) would allow us to identify significant ANOVA differences (ES > 0.187; Critical F = 3.867) with a power of 0.95 and an alpha level of 0.05. Anthropometric, body composition, and fitness characteristics of the sample are shown in Table 1. Results from between-group means comparison are shown in Table 2. Table tennis players presented disparities in anthropometry and body composition compared to physically active children by means of lower BMI (MD (95% CI)= −0.1 to −1.3 kg·m^−2^, ES = 0.24 in boys; −0.6 to −1.8 kg·m^−2^, ES = 0.42 in girls), greater calf- muscle perimeter (MD (95% CI)= 0.2 to 1.4 cm, ES = 0.26 in boys; <0.1 to 1.3 cm, ES = 0.19 in girls), larger bone diameters (MD (95% CI)= 0.01 to 0.05 mm, ES = 0.25 to 0.91 in boys; 0.01 to 0.03 mm, ES = 0.33 to 0.67 in girls) and greater bone mass (MD (95% CI)= 0.51 to 0.89 kg, ES = 0.74 in boys; 0.59 to 1.01 kg, ES = 0.76 in girls). Sex comparisons revealed a greater presence of fat but lower muscle mass and bone density in girls (Figure 1). Somatotype of table tennis players was centered and predominantly mesomorph, while active children had a greater endomorph tendency (Figure 2).

Table tennis players showed superior fitness levels as compared to physically active children, with greater maximal aerobic capacity (MD (95% CI) = 0.48 to 1.92 mL·kg^−1^·min^−1^, ES = 0.34 in boys; 0.57 to 1.83 mL·kg^−1^·min^−1^, ES = 0.39 in girls) and handgrip strength (MD (95% CI) = 0.87 to 2.33 kg, ES = 0.44 in boys; 1.01 to 2.39 kg, ES = 0.50 in girls). Low-back ROM was considerably altered by sex, with girls showing greater values than boys. In particular, girls from the table tennis group exhibited greater results than physically active comparators (MD (95% CI) = 0.71 to 3.29 cm, ES = 0.32).

## 4. Discussion

This study presented data from the largest cohort of young table tennis players (182 children aged 10–11 years old) examined to date. The main findings revealed that children who regularly played table tennis had greater bone development and superior physical fitness compared to those who were physically active but not engaged in a regular physical activity. These results constitute an important first step in clarifying the effectiveness of table tennis as a health-promotion strategy to encourage children to undertake regular physical activity and limit sedentary behavior.

Table tennis players aged 10–11 years presented higher bone development than physically active children. These results are in line with previous studies suggesting that regular practice of racket sports may induce an osteogenic effect in the arm and forehand [41]. Experienced, adult tennis players are shown to develop greater bone mineral content and density, particularly in the dominant side [41,42]. Previous studies in children suggested that enrollment in sport activities during childhood produce improvements in bone development [43,44]. Accordingly, the results of this study demonstrate a positive osteogenic effect among children aged 10–11. Thus, table tennis may constitute an effective strategy to acquire optimal bone mineral accrual during childhood and reduce the risk of osteoporosis in older ages [45].

In support of the slogan recently adopted by the World Health Organization, Every Move Counts [17], the fact that both physically active children and table tennis players presented similar low fat mass below 20% in boys and 30% in girls reinforces the positive impact of regular or recreational exercise during childhood. Indeed, young, high-level table tennis players presented an even lower fat mass, below 20% [46], suggesting that regular table tennis practice may benefit children in maintaining a healthier body composition. The current sex differences identified agreed with natural disparities at these ages [14,15], with girls reaching important maturational events earlier than boys [47]. Interestingly, regular table tennis players did not present higher muscle mass compared to children who sporadically perform other sports. Nonetheless, the observed muscle mass is among the healthy normative data in both groups [14]. Thus, the lack of differences might be due to the high fitness status of the comparators (physically active children group) rather than a detriment of the table tennis players. On the whole, the engagement of children and adolescents in physical activities and sports that promote muscle-mass development is beneficial to health [48]. In this sense, young competitive table tennis players are shown to develop a notable muscle mass [46]. These findings suggest that while recreational table tennis practice preserves adiposity, a highly competitive practice would be required to induce muscle hypertrophy.

Regular table tennis players exhibited a superior cardiovascular fitness and strength compared to active children. Optimal physical fitness at early ages is a proven biomarker of health status [49]. Previous studies have revealed a highly fit profile among young table tennis players [46] that can be attributed to the explosive nature of table tennis competition, which is characterized by rapid and constant movements [20]. Table tennis players required considerable lower-limb muscle activity [50] in order to perform brief explosive movements, change direction rapidly, and effectively hit the ball during a game [51]. Recent investigations have shown positive short-term adaptations in physical and skills performance after specific strength and ballistic training among table tennis players [52,53]. Our results, however, suggested that recreational table tennis during childhood is not enough to induce notable increments in muscle mass. However, it can be argued that the characteristics of table tennis may enhance motor skills up to the point of increasing the ability to produce force in a ballistic action (i.e., the rate of force development) [53,54,55,56]. Future studies should confirm this hypothesis by introducing specific ballistic tests [56] to determine whether table tennis practice would induce more efficient motor-unit recruitment and force twitches in short time periods.

Considering racket sports such as table tennis as an adequate alternative for health promotion, it is important to also consider that prolonged racket sports practice may lead to chronic body asymmetries due to its unilateral nature [57,58,59,60]. These asymmetries start to be noticeable after prepuberal ages in high-demanding sports such as tennis [61]. Therefore, young players who have a regular practice should incorporate compensatory exercise to minimize asymmetries and injuries in the long run [62]. It is worth noting that in the data collection of the present investigation, all anthropometric measurements were performed on the left side of the body, which could mitigate the effect of data suggesting a possible asymmetry resulting from the practice of table tennis. Nonetheless, to the best of our knowledge, the effect of regular table tennis practice on body asymmetries has not been examined yet and merits further attention.

This work has some important strengths, such as the size of sample used and the novelty of the data. However, this study also has certain limitations. The associations identified should be interpreted as exploratory given the cross-sectional nature of the study; thus, it does not allow conclusions about the causal relationships to be drawn. Another potential limitation is that this study did not consider biological maturation, its relationship with sedentary behavior and maturation status in children [63], and the existing sex differences [47]. Future longitudinal and experimental studies are needed to confirm the potential ability of table tennis to improve health among children.

## 5. Conclusions

Regularly played table tennis was associated with superior bone development and physical fitness in children aged 10–11 years compared with physically active controls. However, table tennis practice at these early stages produced no extra benefits in muscle mass compared to the group of active children. This is the largest study to date presenting data about the potential of table tennis to benefit health in children. These results support the effectiveness of table tennis as an enjoyable and accessible activity to promote health among children and limit sedentary behavior.

## Figures and Tables

**Table 1:** Anthropometric, body composition, and fitness characteristics of the sample.

Variable	Table Tennis				Physically Active			
	Boys		Girls		Boys		Girls	
	M ± SD	Range	M ± SD	Range	M ± SD	Range	M ± SD	Range
Anthropometry								
Height (m)	1.44 ± 0.06	1.31–1.57	1.45 ± 0.09	1.23–1.69	1.40 ± 0.08	1.22–1.56	1.39 ± 0.08	1.20–1.56
Weight (kg)	37.1 ± 6.0	26.4–53.1	37.4 ± 7.4	23.2–56.6	36.4 ± 9.2	21.0–62.0	37.1 ± 9.4	22.0–64.0
BMI (kg·m^−2^)	17.8 ± 2.1	14.6–23.7	17.7 ± 2.2	13.2–22.7	18.5 ± 3.6	13.1–32.0	18.9 ± 3.4	12.3–29.7
Skinfolds								
Biceps (mm)	6.8 ± 2.9	2.4–15.2	7.4 ± 3.7	3.0–17.2	6.7 ± 3.8	1.0–18.2	7.7 ± 3.5	2.4–20.4
Triceps (mm)	12.9 ± 4.5	5.0–26.0	14.2 ± 4.9	8.0–27.0	12.9 ± 5.7	4.0–28.0	14.8 ± 5.0	5.0–27.0
Subscapular (mm)	8.1 ± 3.1	3.6–16.3	9.5 ± 5.0	4.0–23.8	8.51 ± 5.9	3.2–29.0	9.7 ± 4.9	3.6–26.0
Suprailiac (mm)	7.9 ± 3.7	30.0–21.1	8.7 ± 4.4	3.0–24.7	7.4 ± 4.9	1.6–24.6	8.9 ± 4.9	2.8–24.4
Abdominal (mm)	12.9 ± 7.3	3.9–33.3	13.3 ± 6.5	4.3–31.6	12.6 ± 8.5	3.4–36.4	14.9 ± 7.7	3.2–37.2
Thigh (mm)	21.0 ± 7.3	6.0–39.0	20.9 ± 6.4	10.0–42.0	18.4 ± 8.5	4.0–43.0	22.1 ± 7.0	7.0–39.0
Medial calf (mm)	14.1 ± 5.5	5.2–29.6	14.5 ± 5.6	7.2–38.8	13.8 ± 7.4	3.0–38.0	16.9 ± 6.0	5.0–30.0
∑6 skinfolds (mm)	76.9 ± 28.6	31.1–140.1	81.2 ± 29.7	46.5–163.0	73.7 ± 38.8	22.4–177.8	87.3 ± 32.4	32.6–169.6
∑trunk skinfolds (mm)	28.9 ± 13.3	12.7–64.1	31.6 ± 15.1	13.9–72.4	28.6 ± 18.6	10.2–86.6	33.5 ± 16.9	9.6–79.6
Perimeters								
Arm relaxed (cm)	22.2 ± 2.3	19.0–27.0	22.4 ± 2.5	17.6–28.0	22.4 ± 2.5	17.6–28.0	22.9 ± 3.1	16.8–30.6
Calf (cm)	29.9 ± 2.6	24.0–35.4	30.2 ± 2.9	22.6–37.0	29.1 ± 3.5	22.5–37.2	29.6 ± 3.5	21.4–37.5
Bone diameters								
Wrist (mm)	4.7 ± 0.3	4.0–5.5	4.6 ± 0.3	3.8–5.5	4.5 ± 0.2	3.7–5.2	4.4 ± 0.3	3.9–5.9
Femur (mm)	8.8 ± 0.5	7.8–10.0	8.4 ± 0.4	7.4–9.5	8.3 ± 0.6	5.5–9.8	8.1 ± 0.5	5.4–9.8
Humerus (mm)	5.7 ± 0.4	4.8–8.7	5.6 ± 0.3	4.9–6.6	5.6 ± 0.4	4.6–6.7	5.5 ± 0.3	4.7–6.8
Body composition								
Muscular (kg)	16.2 ± 2.4	11.7–23.8	15.7 ± 2.8	9.8–22.3	16.3 ± 3.7	9.1–27.3	15.9 ± 3.6	10.1–26.5
Fat (kg)	4.6 ± 1.7	2.4–9.4	6.8 ± 2.7	3.1–16.1	4.7 ± 2.7	1.7–13.5	7.1 ± 3.4	2.9–19.3
Bone (kg)	7.3 ± 0.9	5.5–9.8	7.1 ± 1.1	5.1–10.7	6.6 ± 1.0	4.2–8.9	6.3 ± 1.0	3.9–9.3
Muscular (%)	43.8 ± 1.9	39.2–47.5	42.3 ± 2.9	32.9–46.7	45.0 ± 2.2	38.9–52.6	43.3 ± 3.0	34.4–51.7
Fat (%)	12.2 ± 2.6	8.8–18.1	17.7 ± 4.1	13.0–28.5	12.1 ± 3.7	8.1–22.6	18.2 ± 4.5	11.1–30.2
Bone (%)	16.2 ± 2.4	11.7–23.8	15.7 ± 2.8	9.8–22.3	16.3 ± 3.7	9.1–27.3	15.9 ± 3.6	10.1–26.5
Somatotype								
Endomorph	3.1 ± 1.2	0.9–5.9	3.4 ± 1.4	1.6–7.0	3.4 ± 1.7	0.9–8.1	4.0 ± 1.5	1.3–7.1
Mesomorph	4.3 ± 1.2	0.6–6.8	4.0 ± 1.1	1.0–6.6	4.5 ± 1.2	2.0–8.5	4.4 ± 1.0	1.6–6.8
Ectomorph	3.2 ± 1.2	0.7–6.0	3.3 ± 1.4	0.2–7.7	2.6 ± 1.4	0.1–5.7	2.3 ± 1.5	0.1–7.0
Physical fitness								
VO_2max_ (mL·kg^−1^·min^−1^)	47.8 ± 3.4	39.6–56.9	45.5 ± 2.7	39.6–54.4	46.6 ± 3.7	38.8–54.9	44.3 ± 3.5	34.7–51.1
VO_2max_ (L·min^−1^)	1.77 ± 0.29	1.18–2.84	1.70 ± 0.35	1.01–2.54	1.67 ± 0.37	0.87–2.72	1.62 ± 0.35	0.85–2.45
Low-back ROM (cm)	16.2 ± 4.8	7.0–30.0	20.7 ± 7.0	5.0–38.0	16.7 ± 5.8	3.0–37.0	18.7 ± 5.5	3.0–32.0
Handgrip strength (kg)	18.7 ± 3.5	9.1–27.8	17.1 ± 2.8	11.0–22.8	17.1 ± 3.7	10.5–27.0	15.4 ± 3.9	8.0–28.5

BMI: body mass index. VO_2max_: maximal oxygen consumption. ROM: range of motion.

**Table 2:** Means difference (MD) between table tennis players and physically active children.

Variable	Between-Group Differences				ANOVA *p*-Value		
	Boys		Girls		Group	Sex	Sex × Group
	MD	95% CI	MD	95% CI			
Anthropometry							
Height (m)	0.04	−0.10; 0.18	0.06	−0.11; 0.23	<0.001 *	0.726	0.718
Weight (kg)	0.60	−0.96; 2.16	0.30	−1.41; 2.01	0.554	0.541	0.838
BMI (kg·m^−2^)	−0.70	−1.29; −0.11	−1.20	−1.78; −0.62	0.002 *	0.533	0.416
Skinfolds							
Biceps (mm)	0.10	−0.94; 1.14	−0.60	−1.61; 0.41	0.731	0.022 *	0.601
Triceps (mm)	−0.40	−1.34; 0.54	−0.20	−1.21; 0.81	0.633	0.003 *	0.538
Subscapular (mm)	0.50	−0.38; 1.38	−0.20	−1.15; 0.75	0.544	0.008 *	0.759
Suprailiac (mm)	0.30	−1.31; 1.91	−1.60	−3.04; −0.16	0.787	0.013 *	0.535
Abdominal (mm)	2.60	0.97; 4.23	−1.20	−2.56; 0.16	0.402	0.095	0.227
Thigh (mm)	0.30	−1.02; 1.62	−2.40	−3.58; −1.22	0.377	0.019 *	0.013 *
Medial calf (mm)	3.20	−3.69; 10.09	−6.10	−12.41; 0.21	0.109	0.007 *	0.041 *
∑6 skinfolds (mm)	0.30	−2.96; 3.56	−1.90	−5.15; 1.35	0.671	0.009 *	0.171
∑trunk skinfolds (mm)	0.10	−0.94; 1.14	−0.60	−1.61; 0.41	0.617	0.024 *	0.514
Perimeters							
Arm relaxed (cm)	−0.20	−0.69; 0.29	−0.50	−1.07; 0.07	0.260	0.238	0.668
Calf (cm)	−0.20	−0.69; 0.29	−0.50	−1.07; 0.07	0.023 *	0.257	0.775
Bone diameters							
Wrist (mm)	0.02	0.01; 0.03	0.02	0.01; 0.03	<0.001 *	0.001 *	0.445
Femur (mm)	0.05	0.04; 0.06	0.03	0.02; 0.04	<0.001 *	<0.001 *	0.301
Humerus (mm)	0.01	0.01; 0.02	0.01	0.01; 0.02	<0.001 *	<0.001 *	0.539
Body composition							
Muscular (kg)	−0.10	−0.73; 0.53	−0.20	−0.85; 0.45	0.651	0.222	0.865
Fat (kg)	−0.10	−0.55; 0.35	−0.30	−0.92; 0.32	0.550	<0.001 *	0.709
Bone (kg)	0.70	0.51; 0.89	0.80	0.59; 1.01	<0.001 *	0.010 *	0.743
Muscular (%)	−1.20	−1.62; −0.78	−1.00	−1.60; −0.40	<0.001 *	<0.001 *	0.794
Fat (%)	0.10	−0.55; 0.75	−0.50	−1.37; 0.37	0.537	<0.001 *	0.456
Bone (%)	−0.10	−0.73; 0.53	−0.20	−0.85; 0.45	<0.001 *	<0.001 *	0.408
Somatotype							
Endomorph	−0.30	−0.60; 0.01	−0.60	−0.89; −0.31	0.002 *	0.004 *	0.239
Mesomorph	−0.20	−0.44; 0.04	−0.40	−0.61; −0.19	0.026 *	0.067	0.372
Ectomorph	0.60	0.34; 0.86	1.00	0.71; 1.29	<0.001 *	0.403	0.202
Physical fitness							
VO_2max_ (mL·kg^−1^·min^−1^)	1.20	0.48; 1.92	1.20	0.57; 1.83	0.001 *	<0.001 *	0.903
VO_2max_ (L·min^−1^)	0.10	0.03; 0.17	0.08	0.01; 0.15	0.021 *	0.103	0.814
Low-back ROM (cm)	−0.50	−1.58; 0.58	2.00	0.71; 3.29	0.184	<0.001 *	0.038 *
Handgrip strength (kg)	1.60	0.87; 2.33	1.70	1.01; 2.39	<0.001 *	<0.001 *	0.838

* Significant differences (*p* < 0.05). BMI: body mass index. VO_2max_: maximal oxygen consumption.

## Data Availability

The data presented in this study are available on request from the corresponding author. The data are not publicly available due to privacy.
